# A window of opportunity trial evaluating intratumoral injection of Copaxone® in patients with percutaneously accessible tumors

**DOI:** 10.1186/s41231-023-00137-9

**Published:** 2023-02-27

**Authors:** Joaquina C. Baranda, Ghulam Rehman Mohyuddin, Andrés M. Bur, Yelizaveta Shnayder, Kyle R. Sweeney, Kiran Kakarala, Megan Prouty, Harsh Pathak, Rajni Puri, Amrita Mitra, Rashna Madan, M. Laird Forrest, Aric Huang, Scott Weir, Andrew K. Godwin, Nabil A. Alhakamy, J. Daniel Griffin, Cory J. Berkland

**Affiliations:** 1 Clinical Research Center, University of Kansas Comprehensive Cancer Center, Fairway, KS, USA.; 2 Division of Hematology and Hematological Malignancies, Huntsman Cancer Institute, University of Utah, Salt Lake City, UT, USA.; 3 Department of Otolaryngology-Head and Neck Surgery, University of Kansas Medical Center, Kansas City, KS, USA.; 4 Department of Orthopedic Surgery and Sports Medicine, Sarcoma Center, University of Kansas Comprehensive Cancer Center, Kansas City, KS, USA.; 5 Department of Dermatology, University of Utah, Salt Lake City, UT, USA.; 6 Department of Pathology and Laboratory Medicine, Medical Center, University of Kansas, Kansas City, KS, USA.; 7 Kansas Institute for Precision Medicine, Medical Center, University of Kansas, Kansas City, KS, USA.; 8 Department of Pharmaceutical Chemistry, University of Kansas, Lawrence, KS, USA.; 9 Institute for Advancing Medical Innovation, Medical Center, University of Kansas, Kansas City, KS, USA.; 10 Department of Pharmaceutics, Faculty of Pharmacy, King Abdulaziz University, Jeddah, Saudi Arabia.; 11 Center of Excellence for Drug Research and Pharmaceutical Industries, King Abdulaziz University, Jeddah, Saudi Arabia.; 12 Mohamed Saeed Tamer Chair for Pharmaceutical Industries, Faculty of Pharmacy, King Abdulaziz University, Jeddah, Saudi Arabia.; 13 Kinimmune, Inc, St. Louis, MO, USA.; 14 Department of Chemical and Petroleum Engineering, University of Kansas, Lawrence, KS, USA.

## Abstract

**Background:**

This window of opportunity trial evaluated the safety of intratumoral Copaxone® and profiled immune markers in biopsies before and after treatment.

**Methods:**

Patients with percutaneously accessible malignancies scheduled for surgical resection with curative intent were eligible to participate. Adverse events from one, two, or three injections of Copaxone® were monitored leading up to surgical resection. Using RNA sequencing and spatial protein profiling of immune-related targets, changes in mRNA and protein expression patterns, respectively were assessed in tumor biopsy samples pre- and post-treatment.

**Results:**

Adverse events at the injection site were mild and consistent with historic subcutaneous administration of Copaxone®. Increased intratumoral immune activity was evident in most patients, including the upregulation of genes associated with immune stimulation and targets of checkpoint inhibitor therapy.

**Conclusions:**

Intratumoral injection of Copaxone® was well tolerated, and immune profile changes in the tumor microenvironment warrant its further evaluation as human intratumoral immunotherapy.

**Trial registration:**

clinicaltrials.gov, NCT03982212 First posted June 11^th^,2019

## Introduction

Immunotherapies have advanced modern oncology practices. Checkpoint inhibitors (CPIs), for example, have improved patient response rates and survival across multiple cancers [1, 2]. Most solid tumors, however, remain difficult to treat. Positive outcomes are mainly linked to ‘hot’ tumors—those exhibiting at least some immunological activity such as certain tumor-infiltrating lymphocytes [3]. To improve immune responsiveness, human intratumoral immunotherapy (HIT-IT) has been explored both as monotherapy and in combination with CPIs across many clinical trials [3]. Many drugs evaluated in early HIT-IT studies were already approved for other indications before extrapolation to exploratory trials investigating utility in cancer. Our group discovered that the active ingredient in Copaxone® possesses key molecular attributes for retention in tumors and that this function may induce a local inflammatory microenvironment [4–6]. These studies motivated a “window of opportunity” clinical trial exploring Copaxone® as HIT-IT for the first time.

**Fig. 1:**
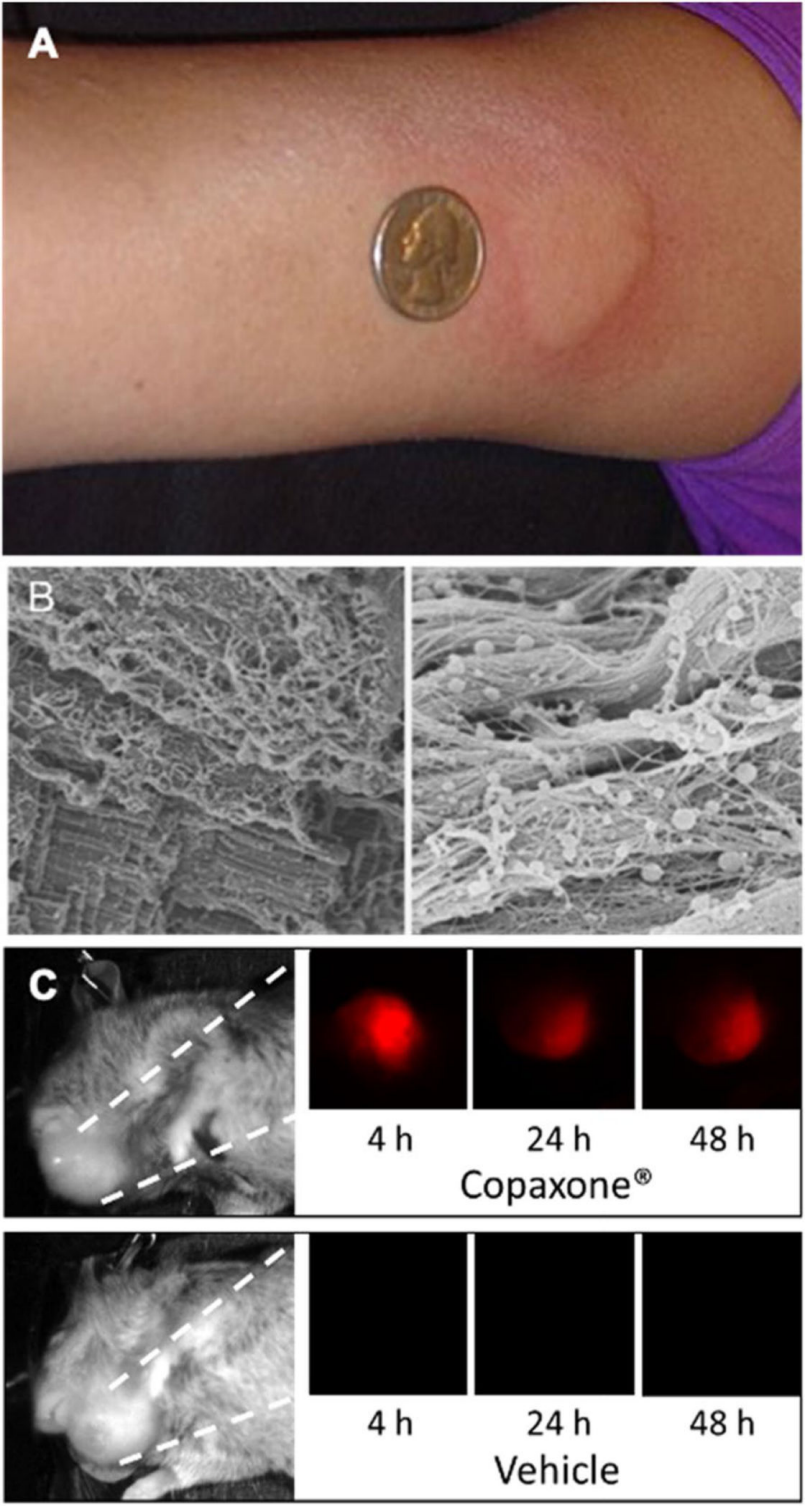
**A** Exemplary injection site reaction after subcutaneous Copaxone® injection. **B** GA precipitates as spherical particles at the injection site. **C** GA labeled with an infrared dye was injected into HNSCC orthotopic tumors in mice and tracked for 48 h. GA persisted in tumors > 2 days while injections of vehicles (4% mannitol) were null

Copaxone® was approved for subcutaneous injection to treat relapsing forms of multiple sclerosis (MS). Notably, injection site reactions are reported in 60–80% of patients (Fig. 1A) [7, 8]. These injection site hallmarks of inflammation, combined with undetectable systemic exposure, prompted our laboratory to investigate the in situ behavior of the active ingredient, glatiramer acetate (GA). Our previous studies found that the mixture of highly cationic peptides in GA precipitated as spherical particles at the injection site and bound to the extracellular matrix (Fig. 1B)4. We demonstrated that GA was retained in AT84 head and neck tumor tissue for over 48 h after intratumoral injection (Fig. 1C) [5].

Our preliminary characterization of the Copaxone® injection site mechanism provided a unique opportunity to quickly advance this drug into a HIT-IT ‘window of opportunity’ clinical trial [4–6]. As safety is of paramount concern in developing new immunotherapies, we endeavored to evaluate Copaxone®, an approved drug with a long-established track record in this regard, in an oncological indication. Patients with percutaneously accessible malignancies who were candidates for standard of care surgical resection with curative intent were eligible to participate. We compared biopsies of tumor tissues to resected samples that had been treated with 1, 2, or 3 Copaxone® injections. In addition to evaluating safety, we probed paired samples using traditional histological staining, RNAseq, and digital spatial cancer immune proteomics analysis (i.e., Nanostring GeoMx® Digital Spatial Profiler) to determine changes in the tumor microenvironment after treatment.

## Materials and methods

### Clinical protocol

#### Study design and patients

This study was an open label single arm window of opportunity trial in patients with resectable solid malignancies whose planned primary treatment was surgical resection conducted at the University of Kansas Comprehensive Cancer Center (KUCCC). The patients had to be at least 18 years old with previously untreated histologically confirmed malignant tumor that was percutaneously accessible for intratumoral injection. The injectable tumor requirement was a size of at least 5 mm in diameter using standard measuring tape. Pretreatment archival tumor tissue consisting of formalin-fixed, paraffin-embedded (FFPE) tumor tissue obtained from standard of care diagnostic biopsy were required to be available to be eligible for the trial. Patients whose lesions were near vascular structures (i.e., carotid artery or tumors close to other vital organs such as the trachea), with mucosal lesions only, with known hypersensitivity to Copaxone® or with a known condition that leads to immunosuppression such as AIDS or concurrent use of immunosuppressive therapy were considered ineligible. Approval for the study was granted through the Institutional Review Board at University of Kansas Cancer Center and all patients provided written informed consent before study entry. This trial was performed according to the Declaration of Helsinki principles. The Kansas University Cancer Center (KUCC) Data and Safety Monitoring Committee (DSMC) performed the oversight of the monitoring of participant safety, conduct and scientific progress of research protocols, and the validity and integrity of the data for clinical trials.

#### Study Treatment

Treatment was administered on an outpatient basis prior to surgery. The pre- to post-treatment window was determined by surgical scheduling and varied between 4–10 days. Glatiramer acetate (Copaxone®) at 40 mg (40 mg/ml) was administered by intratumoral injection, for at least one dose and up to a maximum of three doses prior to surgery. The 40 mg/mL dose strength of Copaxone® was selected for this study because its approved 3-times-per-week dosing frequency is most conducive for the outpatient setting, and likewise resembles that of similar intratumoral trials [9, 10]. 40 mg/mL Copaxone® is also reported to be more tolerable than its more frequently dosed, 20 mg/mL counterpart [11]. Doses were administered at least 48 h apart and the last dose was given within 96 h of surgery. This window of 96 h was chosen because we hypothesized that any immune changes due to Copaxone may remain apparent within 96 hours [8]. No dose adjustments, modifications or delays due to Copaxone® related toxicity were allowed.

#### Injection Technique

After selection of percutaneously accessible tumors, the assigned dose of glatiramer acetate (Copaxone®) was administered using a 28-gauge or smaller needle. Efforts were made to administer the full dose into the lesion unless it was deemed not practicable by the treating investigator. Glatiramer acetate (Copaxone®) was thoroughly distributed within the injected tumor using a “fanning method” (to distribute the injection across several angles throughout the lesion to maximize the spread of glatiramer acetate in the tumor). The use of local anesthetic prior to Copaxone injection was allowed.

#### Safety Assessments

Adverse events were assessed at baseline, during Copaxone® administration, and at the time of surgery or end of treatment visit. The revised NCI Common Terminology Criteria for Adverse Events (CTCAE) version 5.0 was utilized for AE reporting and toxicity assessment. Complete blood count, chemistry and INR were done at baseline.

#### Ki‑67 and Caspase‑3 levels

Changes in the expression levels of Ki-67 and caspase-3 before and after treatment with Copaxone® were evaluated by IHC. FFPE tumor sections from pre- and post-treatment specimens were mounted on the same slide and stained using Caspase 3 (Biocare, Pacheco, CA) and Ki-67 (MIB-1) (Dako, Carpinteria, CA).

### RNA‑Seq Analysis

A cutoff linear fold-change of 4 was used to select the differentially expressed genes in each patient. Gene ontology (GO) of the biological process, functional annotation clustering, and Kyoto Encyclopedia of Genes and Genomes (KEGG) pathway analysis of the differentially expressed genes were performed using Database for Annotation, Visualization and Integrated Discovery (DAVID) v2021 [12, 13]. For groups that had more than 3000 differentially expressed genes (i.e. upregulated gene expression for patients 1 and 3), functional annotation clustering was performed using the top 3000 most differently expressed genes.

### GeoMx® Digital Spatial Profiling Analysis

The GeoMx® Digital Spatial Profiling (DSP) platform (NanoString) allows users to perform nondestructive interrogation of unstained tissue sections (among other tissue types) for multiplexed spatial profiling of immune-oncology-related proteins to assess tumor cells and their microenvironment. The GeoMx® DSP platform uses a cocktail of antibodies conjugated to photocleavable DNA-barcoded oligos to provide high multiplex capacity, and the use of guided ultraviolet light exposure (by way of adjustable 1 million micromirrors) provides a high degree of flexibility in the selection of regions of interest for study. DSP technology and the GeoMx® platform have been extensively reviewed [14–17].

DSP of pre- and post-treatment FFPE tumor tissue was performed to measure the relative levels of 28 protein markers associated with immuno-oncology. Specifically, the immune cell profiling core protein module and IO drug target protein module (Supplementary Table 1) were used for this analysis following the manufacturer’s protocol for staining, hybridization, collection, detection (nCounter based-counting), and data normalization. Briefly, this entailed an initial review of hematoxylin and eosin (H&E)-stained sections by a pathologist at KUMC to verify the presence of tumor cells within the FFPE block (Supplementary Fig. 2). Next, a 5 μm-thick unstained FFPE slide was subjected to a cocktail of primary antibodies conjugated with unique DNA-oligonucleotide moieties attached with a light-sensitive photocleavable linker. Within this cocktail were also anti-pan-cytokeratin (included with GeoMx® assay and used for the basal cell carcinoma and squamous cell carcinoma samples) or anti-MART-1 (Novus Biologicals, catalog # NBP2–46603AF647 used for targeting epithelium in melanoma samples), CD45 (for targeting immune cells), and Syto-13 (used as a nuclear stain). These fluorescently labeled antibodies were used for imaging and identifying the regions of interest (ROIs), followed by segmentation into tumor cells and immune cells for independent collection from each ROI into individual wells of a 96-well plate, followed by digital quantification using the NanoString nCounter platform. The GeoMx® DSP Analysis Suite version 2.4 was used to perform data QC, normalization, and statistical analysis following guidance by NanoString field scientists. For analysis of similar cell populations (*i.e.,* expression levels only in epithelial cells or only in immune cells), the geometric mean of housekeeping proteins GAPDH, Histone H3, and S6 was used for normalization. When different cell populations were being analyzed (*i.e.,* expression levels in epithelial versus immune cells), the geometric mean of the signal-to-noise ratio of a negative control mouse and rabbit isotype IgG controls was used for background correction prior.

## Results and discussion

Although cancer immunotherapies have grown in promise for treating aggressive cancers, activating the immune system against primary solid tumors and perhaps even against secondary metastases (*i.e.,* the ‘Abscopal Effect’) will likely require a multipronged approach [9]. Immune checkpoint inhibitors inhibit negative feedback mechanisms on immune cells and are expected to work synergistically with immunostimulants that promote proinflammatory stimulation inside tumor tissues [3]. In this regard, the intratumoral injection of immunostimulants that persist at the injection site can be advantageous when compared with systemically distributed formulations since side effects and drug-drug interactions may be limited [9, 10]. Multiple clinical trials are currently underway to assess such combination approaches to cancer immunotherapy [9, 10], and the recent acquisitions of Checkmate Pharmaceuticals (Regeneron) and Immune Design (Merck) highlight commercial interest. Understanding that GA has negligible systemic exposure in humans and having confirmed that GA persists in mouse tumors after intratumoral injection (Fig. 1C), this clinical study aimed to establish key clinical data for Copaxone® as a HIT-IT.

We conducted a single-arm, open-label, perioperative window of opportunity trial to evaluate the feasibility, safety, and local effects of intratumoral/peritumoral injections of Copaxone®. Patients whose tumors were accessible for intratumoral, percutaneous injection and were planned for surgery as the primary treatment were eligible and consented. Patients were ineligible for the study if they were scheduled to receive other treatments between the initial biopsy and surgery. During the treatment period, eligible subjects received Copaxone® (40 mg dose) intratumorally up to three times a week and at least 48 h apart until 24 h before the planned surgery (Table 1). This dosing regimen was consistent with the label for subcutaneous injections of Copaxone® at the 40 mg/mL dose strength.

The primary endpoint assessed was adverse events associated with intratumoral/peritumoral injections of Copaxone®. Injection site reactions were observed in and around tumor tissues, like those observed following subcutaneous injections of Copaxone® reported in MS patients [4]. Of the nine patients enrolled, only three treatment-related adverse events were reported. All were Grade 1 (mild) reactions, occurring at the injection site and included itching, pressure, burning sensation, and pain (Table 2). Ki67 expression deceased in each patient where residual tumor was sufficient to conduct the analysis (Table 2). Caspase 3 was consistently at or below 5% with no discernable treatement-related changes.

We assessed immune biomarkers in tumor biopsies and compared them with resected tumors after one, two, or three treatments. Conventional RNA sequencing and spatial protein profiling of 28 mostly immune-related targets (*i.e.,* Nanostring GeoMx® DSP) were used to assess immune markers at the transcriptional and protein levels. In the RNA sequencing analysis, genes that were differentially expressed less than four fold (|FC|< 4) between the pre- and post-treatment samples for three patients were filtered out. For patient 1, 6355 genes were upregulated, and 406 genes were downregulated post-treatment. For patient 2, 647 genes were upregulated, and 75 genes were downregulated post-treatment. For patient 3, 5375 genes were upregulated, and 128 genes were downregulated post-treatment. A complete list of differential gene expression analysis results is included in Supplementary Data File 1.

**Fig. 2:**
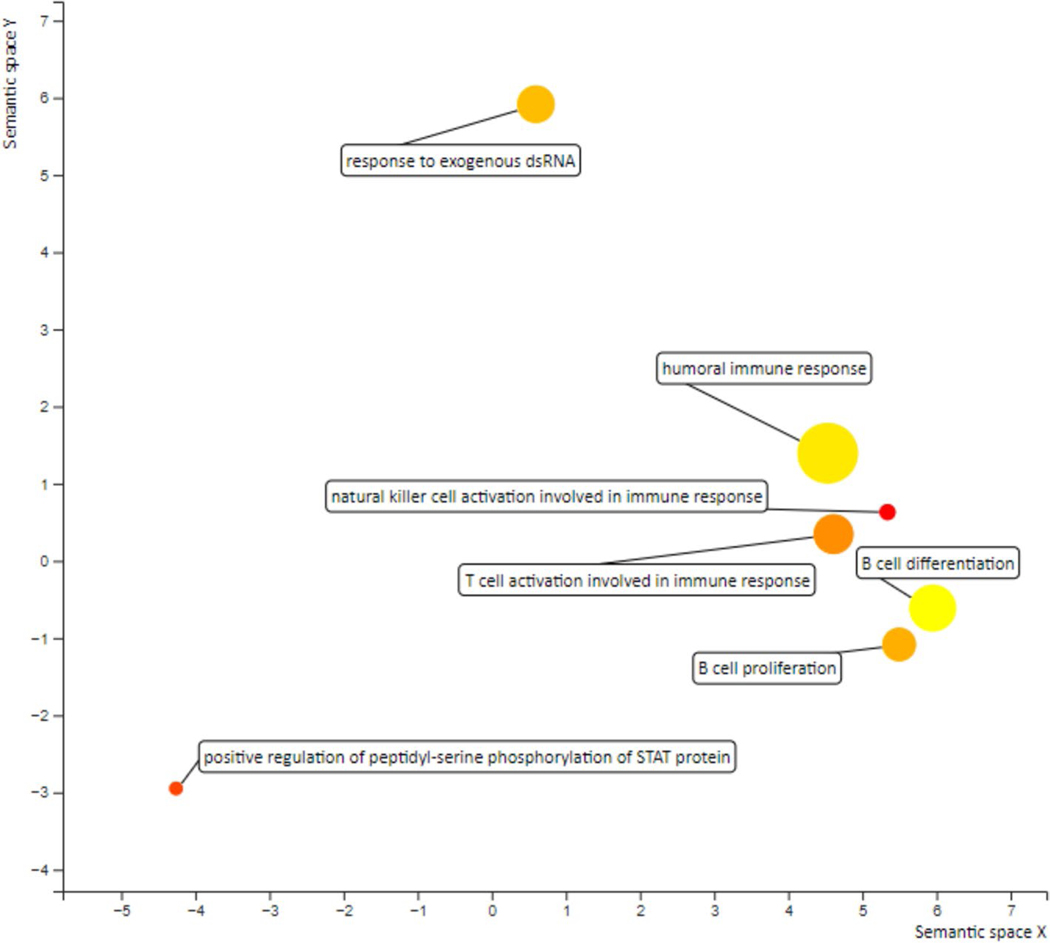
Gene ontology plot generated using RNA sequencing data that was statistically significant for each of patients 1, 2, and 3. Gene families are given for each data point. Color intensity denotes the number and magnitude of expression in each category. The size of the circle indicates the relative breadth of genes expressed in each category (smaller circles = fewer genes expressed in that category). Data points more closely grouped indicate the categories are more closely related

Gene Ontology (GO) enrichment analysis was performed to categorize differential gene expressions into specific biological processes, followed by Functional Annotation Clustering to group-related GO terms (Supplementary Data File 2). All three patients had an upregulation in genes involved in promoting an immune-rich tumor microenvironment, such as NK cell activation, positive regulation of STAT protein, T-cell activation, humoral immune response, response to exogenous dsRNA, and B-cell proliferation and differentiation (Fig. 2). Individual GO plots were also developed for patients 1, 2 and 3 (Supplementary Fig. 1). No categories of biological processes were consistently found to be downregulated across all the three post-treatment patient samples. Though only obtained from three patients, bulk RNA sequencing was used to build conviction around GeoMx spatial analysis methods.

Using the GeoMx DSP platform, *in situ* digital spatial profiling was performed in tissue specimens to measure levels of 28 proteins before and after treatment (Immune cell profiling panel and the IO drug target panel, Supplementary Table 1). After a pathologist review of H&E-stained FFPE tissue specimens to assess the presence of tumor cells, GeoMx® DSP analysis was performed on a serial unstained slide for each sample (Supplementary Figure 2). Regions of interest (ROIs) which encompassed both tumor and immune cells, were selected either by free-hand drawing using the polygon tool or using a standard circle. Supplementary Figure 3A and B show the ROIs selected for all samples. The ROIs were further segmented into tumor cells and immune cells based on the staining pattern of morphology markers, *i.e.* pan-cytokeratin and CD45 for the carcinomas and MART1 and CD45 for the melanomas, respectively.

**Fig. 3:**
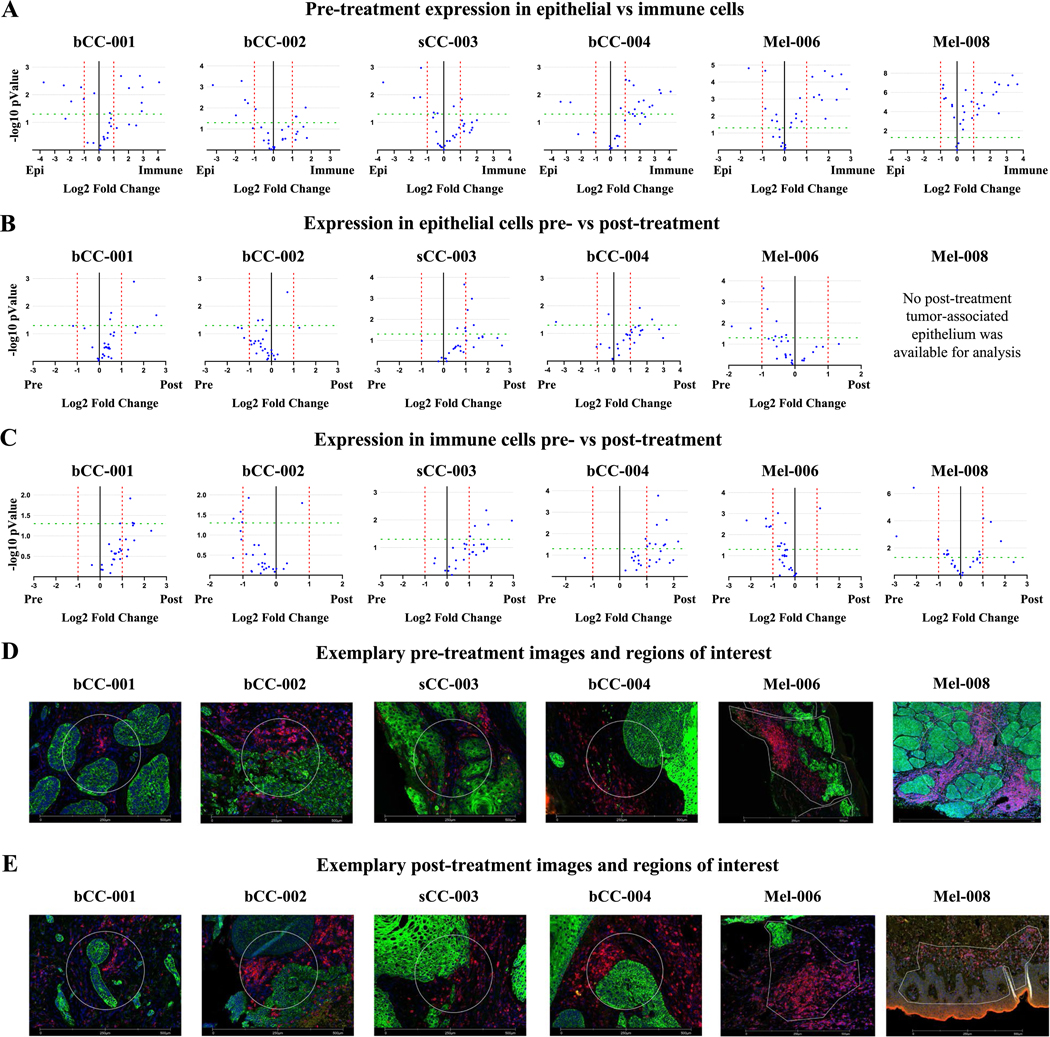
Volcano plots showing differential protein expression. Expression was considered statistically significant if changes were ≥ ± two fold (≥ ± one fold in Log2 scale, red dashed vertical lines) with a p value ≤ 0.05 (≥ 1.3 in-Log10 scale, green dashed horizontal line). A paired t-test was used for these analyses. **A** Comparison of baseline (pre-treatment) expression between epithelial and immune cells. **B** Comparison of expression in epithelial cell before and after treatment. **C** Comparison of expression in immune cells before and after treatment. An excel file is provided for all volcano plots in supplementary Tables 2–4. **D** Representative images used for pre-treatment GeoMx analysis. **E** Representative images used for pre-treatment GeoMx analyses (all images are available in Supplementary Fig. 3A and B). The time elapsed between pre and post treatment analyses was dictated by surgical reaction scheduling and varied between 4–10 days

Following segmentation, the DNA bar codes corresponding to each target in the antibody cocktail were collected by the instrument. The DNA bar codes were processed through the nCounter for digital counting and finally analyzed through the GeoMx® Analysis suite. Initial data analysis of immune cells and tumor cells in pre-treatment tissue showed the expected pattern where most of the immune related targets tended to be expressed at higher levels in the immune cells relative to the tumor cells and the few epithelial targets included in the panels tended to be expressed at higher levels in the tumor cells relative to the immune cells (e.g., pan-cytokeratin, and Ki-67) (Figure 3, Table 3, and Supplementary Tables 2–5). Interestingly, we observed that patients showing a larger decrease in Ki-67 levels (10–25% decrease) following treatment (Table 2, patients bCC-001, sCC-003, and bCC-004) had increased expression for a greater number of immune-related markers in the post-treatment tissue (Table 3, Figure 3B and C) as compared to those patients that showed a smaller decrease in Ki-67 levels (5–6% decrease) following treatment (Table 2 patients bCC-002 and Mel-006). These differences were also reflected in the bulk RNA Sequencing results, where the more pronounced Ki-67 responders (bCC-001, bCC-004) also exhibited greater changes in gene ontology pre- to post-treatment (Supplementary Figure 1). Many of the upregulated markers among immune and epithelial cells alike were checkpoint-associated targets, such as PD1, PD-L1, CTLA-4, LAG3, OX40L, B7-H3, and Tim-3 (Table 3, Figure 3B, C, and Supplementary Tables 3 and 4).

Intratumoral administration of immunostimulants such as pattern recognition receptor (PRR) agonists, cytokines, and oncolytics has been widely investigated to overcome “cold” tumor microenvironments [18–21]. Several clinical-stage programs include TLR9-agonist idutolimod (CMP-001), TLR4-agonist tilsotolimod (IMO-2125), IL12 inducer tavokinogene telseplasmid (TAVO), and oncolytic peptide KKWWKKW-Dip-K-NH2 (LTX-315). Leveraging GA as HIT-IT may be advantageous; however, in that its approved regulatory status and the intratumoral safety profile demonstrated here mean GA may be expeditiously repurposed.

Many intratumoral programs are designed for use in combination with other immunotherapies such as CPIs. Our group is exploring whether cationic properties of GA may work synergistically with CPIs [6]. We have also reported that GA can deliver other HIT-ITs such as CpG and PolyI:C [5]. These complexes persist at the intratumoral site of injection and may represent another compelling HIT-IT approach [5]. The clinical utility of HIT-IT is most likely in metastatic of disease where the prospects of antigenic diversity for immune activation and tumor accessibility for injection may be increased [22]. Future studies characterizing the efficacy and safety of localized intratumoral immunostimulation are warranted, including the quantitative analysis of intratumoral T-cell recruitment. Evidenced by contemporary clinical investigations and a growing consensus, meaningful translational immunotherapies for solid tumors will likely consist of combination approaches that can synergize to recruit, potentiate, and propagate potent anti-tumor immune responses [3, 23].

## Conclusion

Intratumoral injections of Copaxone® were well tolerated, with side effects at the injection site mirroring reported subcutaneous injection site reactions in multiple sclerosis patients. Even though a small number of cancer patients were treated with intratumoral injections of Copaxone® in this window of opportunity trial, a reduction of proliferative index was evident in all analyzed samples. Immune markers, including hallmarks of T-cell recruitment and activation, were upregulated in post-treatment tumor samples. Our findings reveal that the local immunomodulatory effects of GA in the intratumoral injection site in patients are safe and may suggest utility as HIT-IT.

## Supplementary Material

Data File 1

Data File 2

1

## Figures and Tables

**Table 1.** Study medication dosing and Copaxone® treatment schedule.

Patient number	Age	Sex	Race	Diagnosis	Intratumoral Treatments	RNAseq	GeoMx
*1*	73.0	M	White	Basal cell, neck	2	X	X
*2*	64.3	M	White	Basal cell cancer, nose	2	X	X
*3*	66.4	M	White	Squamous cell cancer, right cheek	1	X	X
*4*	77.4	M	White	Basal cell cancer, chest	3		X
*5*	43.1	F	White	Melanoma, ear	2		
*6*	67.5	M	White	Melanoma, scalp	2		X
*7*	79.0	M	White	Squamous cell cancer, scalp	2		
*8*	65.8	M	White	Melanoma, back	2		X
*9*	48.2	M	White	Melanoma, back	2		

**Table 2.** Side effects for each study patient and Ki67 results.

Patient number	Diagnosis	Injection Site Reaction	Pre Ki67 results	Post Ki67 results
*1*	Basal cell, neck	Grade 1-Mild (Itching/Pressure)	70%	45%
*2*	Basal cell cancer, nose	Grade 1-Mild (Burning Sensation)	65%	60%
*3*	Squamous cell cancer, right cheek	None	55%	45%
*4*	Basal cell cancer, chest	Injection site pain for 2 hours for first treatment.	62%	44%
*5*	Melanoma, ear	None	No paired tissue	No residual tumor in resection specimen
*6*	Melanoma, scalp	None	9%	3%
*7*	Squamous cell cancer, scalp	NA*		NA*
*8*	Melanoma, back	None	22%	6%
*9*	Melanoma, back	None	No paired tissue	No residual tumor in resection specimen

**Table 3.** Genes upregulated >2-fold when comparing pre-treatment to post-treatment using GeoMx immuno-oncology markers. Immune checkpoint markers are italicized in bold text.

Patient identifier	GeoMx Patient identifier	Diagnosis	Intratumoral Treatments	Genes upregulated >2-fold	
				Immune Cells	Epithelial Cells
*1*	*bCC-001*	Basal cell, neck	2	CD11c, GITR, OX40L, PD-1, GZMB, B7-H3, LAG3, ARG1, CD68	CD56, B7-H3, PD-L1, Ki-67
*2*	*bCC-002*	Basal cell cancer, nose	2	None	GITR
*3*	*sCC-001*	Squamous cell cancer, right cheek	1	CD8, OX40L, CD45, CD4, GZMB, CD11c, CD3, B7-H3, CD56, ARG1, CTLA4, HLA-DR, SMA, CD20, Tim-3	OX40L, SMA, B7-H3, CD45, PD-L1, CD8, CD11c, CD68, GZMB, CD4, HLA-DR
*4*	*bCC-004*	Basal cell cancer, chest	3	ARG1, 4–1BB, IDO1, CD11c, PD-L1, CD56, CTLA4, Beta-2-microglobulin, CD45, GITR, CD3, Fibronectin, HLA-DR, Tim-3, OX40L, CD4	4–1BB, CD56, B7-H3, HLA-DR, Tim-3, CD45, Fibronectin, ARG1, VISTA, PD-L1, STING, GITR
*6*	*Mel-006*	Melanoma, scalp	2	Ki-67	CTLA4
*8*	*Mel-008*	Melanoma, back	2	PanCk, Fibronectin, B7-H3, Beta-2-microglobulin	(Not able to detect)

## Data Availability

All data generated or analyzed during this study are included in this published article and its supplementary information files.
