# Supplementary material for: A window of opportunity trial evaluating intratumoral injection of Copaxone® in patients with percutaneously accessible tumors
Source: Transl Med Commun. Author manuscript; Available in PMC 2025 Apr 14. (PMC11996204; doi:10.1186/s41231-023-00137-9)
Supplement: 1 [file NIHMS1967122-supplement-1.pdf]

Supplementary Tables

Supplementary Table 1. List of targets used in the GeoMx analysis.

	Module	***Target group membership/s***	***Target name***
1	IO Drug Target Module	T cells, T cell Activation	4-1BB
2	IO Drug Target Module	Myeloid Suppression, M2 Macrophage, Myeloid Activation, Macrophage, Myeloid	ARG1
3	IO Drug Target Module	Checkpoint	B7-H3
4	Immune Cell Profiling Core	Antigen Presentation, Tumor	Beta-2-microglobulin
5	Immune Cell Profiling Core	DC, Myeloid	CD11c
6	Immune Cell Profiling Core	B cells	CD20
7	Immune Cell Profiling Core	T cells	CD3
8	Immune Cell Profiling Core	Myeloid, Th cells, T cells	CD4
9	Immune Cell Profiling Core	Total Immune	CD45
10	Immune Cell Profiling Core	NK cells	CD56
11	Immune Cell Profiling Core	M2 Macrophage, Macrophage, Myeloid	CD68
12	Immune Cell Profiling Core	T cells, CD8 T cells	CD8
13	Immune Cell Profiling Core	Checkpoint, Th cells, T cells, T cell Activation	CTLA4
14	Immune Cell Profiling Core	Stroma, Fibroblasts	Fibronectin
15	Immune Cell Profiling Core	Housekeepers	GAPDH
16	IO Drug Target Module	T cells, T cell Activation	GITR
17	Immune Cell Profiling Core	Cytotoxicity, T cell Activation	GZMB
18	Immune Cell Profiling Core	Housekeepers	Histone H3
19	Immune Cell Profiling Core	MHC2, Antigen Presentation	HLA-DR
20	IO Drug Target Module	Myeloid Suppression, Myeloid	IDO1
21	Immune Cell Profiling Core	Proliferation	Ki-67
22	IO Drug Target Module	Checkpoint, T cells, T cell Activation	LAG3
23	Immune Cell Profiling Core	Background negative control	Ms IgG1
24	Immune Cell Profiling Core	Background negative control	Ms IgG2a
25	IO Drug Target Module	Myeloid Activation, T cell Activation	OX40L
26	Immune Cell Profiling Core	Tumor, Epithelial	PanCk
27	Immune Cell Profiling Core	Checkpoint, T cells, T cell Activation	PD-1
28	Immune Cell Profiling Core	Myeloid Activation, Checkpoint	PD-L1
29	Immune Cell Profiling Core	Background negative control	Rb IgG
30	Immune Cell Profiling Core	Housekeepers	S6
31	Immune Cell Profiling Core	Stroma	SMA
32	IO Drug Target Module	Interferon	STING
33	IO Drug Target Module	Checkpoint, T cells, T cell Activation	Tim-3
34	IO Drug Target Module	Myeloid Activation, Checkpoint, Macrophage, Myeloid	VISTA

Supplementary Tables

Supplementary Table 2. GeoMx analysis data of epithelial markers versus immune markers in pretreatment tissue.

bCC-001			bCC-002			sCC-003			bCC-004			Mel-006			Mel-008		
**Target name**	**Log2 fold change**	**-log10 pvalue**	**Target name**	**Log2 fold change**	**-log10 pvalue**	**Target name**	**Log2 fold change**	**-log10 pvalue**	**Target name**	**Log2 fold change**	**-log10 pvalue**	**Target name**	**Log2 fold change**	**-log10 pvalue**	**Target name**	**Log2 fold change**	**-log10 pvalue**
CD45	4.083	2.453	IDO1	1.766	0.573	Fibronectin	2.004	1.078	CD45	4.087	2.116	CD3	2.820	3.580	CD3	3.646	6.841
CD3	2.955	2.272	VISTA	1.703	2.018	SMA	1.732	0.810	VISTA	3.421	2.056	CD45	2.576	4.453	CD45	3.355	7.774
HLA-DR	2.903	1.407	HLA-DR	1.584	1.106	CD45	1.704	1.002	HLA-DR	3.331	2.177	CD4	2.362	2.958	CD4	3.091	6.745
VISTA	2.897	1.711	CTLA4	1.273	0.872	CD3	1.679	0.726	CD3	2.846	1.611	CD8	2.168	4.342	CD8	2.546	6.829
CD4	2.777	2.684	CD3	1.221	1.590	IDO1	1.573	0.630	CD4	2.657	1.558	IDO1	1.859	1.841	IDO1	2.520	3.826
IDO1	2.527	0.890	GITR	1.210	0.762	HLA-DR	1.244	0.949	4-1BB	2.360	1.200	HLA-DR	1.829	4.646	HLA-DR	2.382	6.046
CD8	2.043	0.925	CD45	1.189	1.591	CD4	1.224	0.675	Fibronectin	2.258	1.797	CD11c	1.694	2.940	VISTA	2.301	7.044
PD-L1	2.035	2.243	CD11c	1.168	0.482	PD-L1	1.182	0.873	CD8	2.128	1.333	VISTA	1.571	3.246	CD11c	2.192	5.772
Tim-3	1.498	2.679	STING	0.794	1.113	CD68	1.100	1.073	LAG3	2.012	0.942	Fibronectin	1.331	3.110	CTLA4	1.642	4.628
SMA	1.207	0.795	ARG1	0.631	0.539	4-1BB	1.092	1.837	IDO1	1.926	1.745	CD68	1.261	4.309	CD56	1.501	5.945
CD68	0.820	0.861	CD4	0.497	0.529	GITR	0.928	0.812	GITR	1.659	1.230	GITR	0.774	1.680	Tim-3	1.318	4.325
STING	0.810	1.145	4-1BB	0.486	0.484	VISTA	0.757	0.562	Tim-3	1.653	1.476	CTLA4	0.703	3.057	PD-L1	1.230	3.674
B7-H3	0.737	1.341	Fibronectin	0.455	0.953	CTLA4	0.654	0.456	CD68	1.573	2.343	CD56	0.523	2.125	CD68	1.227	4.780
Fibronectin	0.708	0.985	Beta-2-microglobulin	0.058	0.137	LAG3	0.603	1.583	CTLA4	1.516	1.305	Tim-3	0.520	1.864	Fibronectin	0.907	3.326
CD11c	0.600	0.619	CD20	0.034	0.069	CD8	0.559	0.513	STING	1.276	2.507	4-1BB	0.309	1.605	Ki-67	0.877	5.036
4-1BB	0.580	0.436	CD8	-0.011	0.148	CD11c	0.362	0.327	GZMB	1.249	1.365	CD20	0.281	1.341	GZMB	0.440	3.940
CD20	0.363	0.382	LAG3	-0.101	0.019	ARG1	0.198	0.326	OX40L	1.049	2.545	PD-1	0.037	0.128	GITR	0.375	3.137
CTLA4	0.353	0.406	CD68	-0.103	0.034	B7-H3	0.057	0.157	PD-L1	0.981	1.068	SMA	0.031	0.297	4-1BB	0.363	2.138
GITR	0.313	0.478	PD-L1	-0.208	0.075	PD-1	0.007	0.011	CD11c	0.697	1.516	STING	-0.002	0.086	CD20	0.359	4.572
ARG1	0.103	0.022	GZMB	-0.249	0.462	GZMB	-0.176	0.121	ARG1	0.604	0.489	LAG3	-0.089	0.415	PD-1	0.196	2.716
Beta-2-microglobulin	0.093	0.162	SMA	-0.259	0.309	OX40L	-0.233	0.165	B7-H3	0.498	0.145	OX40L	-0.151	0.220	Beta-2-microglobulin	0.091	0.705
OX40L	0.048	0.016	Tim-3	-0.281	0.805	Tim-3	-0.357	0.227	PD-1	0.477	0.519	Ki-67	-0.224	0.762	LAG3	0.008	0.006
LAG3	-0.372	0.255	PD-1	-0.504	0.455	Beta-2-microglobulin	-0.374	1.328	Beta-2-microglobulin	0.414	0.512	GZMB	-0.227	1.209	OX40L	-0.042	0.360
PD-1	-0.774	0.263	CD56	-0.640	1.077	CD56	-0.454	0.660	CD20	-0.040	0.030	ARG1	-0.340	1.686	PanCk	-0.170	3.382
GZMB	-1.210	2.270	OX40L	-1.343	2.226	CD20	-0.605	1.356	CD56	-1.128	0.635	Beta-2-microglobulin	-0.373	2.087	ARG1	-0.296	4.547
Ki-67	-2.273	1.131	B7-H3	-1.677	3.282	STING	-0.640	1.175	Ki-67	-2.192	0.571	PanCk	-0.515	0.780	STING	-0.302	4.715
CD56	-2.387	2.340	Ki-67	-1.977	1.647	Ki-67	-1.797	1.888	SMA	-2.722	1.725	PD-L1	-0.869	2.433	B7-H3	-0.676	5.418
PanCk	-3.752	2.449	PanCk	-3.196	3.084	PanCk	-3.662	2.450	PanCk	-3.395	1.748	B7-H3	-1.624	4.811	SMA	-0.780	5.331

Supplementary Tables

Supplementary Table 3. GeoMx analysis data of pre- versus post-treatment epithelial markers.

bCC-001			bCC-002			sCC-003			bCC-004			Mel-006			Mel-008		
**Target name**	**Log2 fold change**	**-log10 pvalue**	**Target name**	**Log2 fold change**	**-log10 pvalue**	**Target name**	**Log2 fold change**	**-log10 pvalue**	**Target name**	**Log2 fold change**	**-log10 pvalue**	**Target name**	**Log2 fold change**	**-log10 pvalue**	**Target name**	**Log2 fold change**	**-log10 pvalue**
CD56	2.579	1.673	GITR	1.262	1.218	OX40L	2.641	0.759	4-1BB	2.974	0.834	CTLA4	1.327	1.008	GZMB	n/a	n/a
B7-H3	1.720	1.253	GZMB	0.721	2.508	SMA	2.435	1.116	CD56	2.734	1.152	B7-H3	0.846	0.873	PD-1	n/a	n/a
PD-L1	1.627	1.037	OX40L	0.278	0.251	B7-H3	1.767	1.150	B7-H3	2.314	1.508	Tim-3	0.651	0.872	CD45	n/a	n/a
Ki-67	1.566	2.890	STING	0.163	0.079	CD45	1.758	1.196	HLA-DR	1.725	1.126	Beta-2-microglobulin	0.551	0.621	CD3	n/a	n/a
CD4	0.669	1.054	Fibronectin	0.103	0.213	PD-L1	1.596	1.109	Tim-3	1.628	1.096	PanCk	0.284	0.318	PD-L1	n/a	n/a
CTLA4	0.641	0.928	LAG3	0.016	0.281	CD8	1.364	1.726	CD45	1.565	0.945	GITR	0.271	0.224	SMA	n/a	n/a
SMA	0.544	0.113	IDO1	-0.003	0.413	CD11c	1.361	0.679	Fibronectin	1.565	1.923	PD-L1	0.222	0.231	Ki-67	n/a	n/a
CD11c	0.536	1.760	CD56	-0.126	0.212	CD68	1.283	2.980	ARG1	1.506	1.280	CD11c	-0.074	0.060	CD68	n/a	n/a
PD-1	0.508	0.984	PD-1	-0.154	0.105	GZMB	1.089	2.444	VISTA	1.298	1.223	SMA	-0.087	0.228	CD11c	n/a	n/a
GZMB	0.490	1.527	CTLA4	-0.171	0.308	CD4	1.034	0.988	PD-L1	1.242	0.664	LAG3	-0.102	0.119	CD56	n/a	n/a
CD8	0.473	0.461	CD3	-0.193	0.069	HLA-DR	1.001	1.442	STING	1.093	1.041	OX40L	-0.142	0.500	PanCk	n/a	n/a
OX40L	0.432	0.509	CD68	-0.232	1.112	GITR	0.979	1.585	GITR	1.046	1.137	PD-1	-0.211	1.143	HLA-DR	n/a	n/a
ARG1	0.414	0.168	VISTA	-0.293	0.452	CD3	0.937	3.659	CD4	0.977	0.942	GZMB	-0.264	1.432	CD20	n/a	n/a
IDO1	0.409	0.617	CD8	-0.388	0.805	IDO1	0.902	0.443	IDO1	0.974	0.443	4-1BB	-0.271	0.368	Beta-2-microglobulin	n/a	n/a
CD3	0.407	0.472	Beta-2-microglobulin	-0.398	0.396	CTLA4	0.771	0.499	OX40L	0.969	0.817	CD68	-0.277	0.456	CTLA4	n/a	n/a
CD45	0.334	0.508	Ki-67	-0.402	1.505	Beta-2-microglobulin	0.754	0.713	CTLA4	0.828	0.944	CD20	-0.287	0.518	CD8	n/a	n/a
CD20	0.299	0.524	CD4	-0.484	0.530	Tim-3	0.656	0.753	CD3	0.752	0.974	CD56	-0.400	1.342	Fibronectin	n/a	n/a
Beta-2-microglobulin	0.235	0.655	HLA-DR	-0.488	0.648	Fibronectin	0.624	1.583	LAG3	0.587	0.753	Fibronectin	-0.410	1.134	CD4	n/a	n/a
VISTA	0.232	0.497	B7-H3	-0.585	0.745	LAG3	0.555	0.709	PanCk	0.311	0.213	VISTA	-0.544	0.309	LAG3	n/a	n/a
Fibronectin	0.226	0.203	PanCk	-0.607	1.484	PanCk	0.514	0.646	Beta-2-microglobulin	0.256	0.451	HLA-DR	-0.551	0.887	4-1BB	n/a	n/a
4-1BB	0.161	0.260	CD20	-0.677	0.290	CD56	0.447	0.597	GZMB	0.221	0.611	CD8	-0.557	2.083	B7-H3	n/a	n/a
CD68	0.129	0.129	CD45	-0.730	0.735	Ki-67	0.244	0.397	CD68	-0.070	0.064	ARG1	-0.590	1.370	Tim-3	n/a	n/a
HLA-DR	-0.023	0.086	ARG1	-0.783	0.651	4-1BB	0.140	0.010	CD11c	-0.077	0.086	CD4	-0.640	1.189	OX40L	n/a	n/a
STING	-0.038	0.022	PD-L1	-0.904	0.612	PD-1	0.110	0.134	CD8	-0.265	0.482	CD45	-0.794	1.256	GITR	n/a	n/a
Tim-3	-0.076	0.105	4-1BB	-0.940	0.746	VISTA	0.065	0.141	CD20	-0.314	1.695	CD3	-0.827	2.657	STING	n/a	n/a
GITR	-0.316	0.511	CD11c	-1.236	0.853	CD20	-0.088	0.179	PD-1	-0.793	0.611	STING	-0.950	3.647	IDO1	n/a	n/a
LAG3	-0.672	1.198	Tim-3	-1.342	1.203	ARG1	-0.234	0.006	Ki-67	-1.038	0.514	IDO1	-1.340	1.746	ARG1	n/a	n/a
PanCk	-1.189	1.288	SMA	-1.516	1.226	STING	-0.984	0.975	SMA	-3.483	1.416	Ki-67	-1.909	1.839	VISTA	n/a	n/a

Supplementary Tables

Supplementary Table 4. GeoMx analysis data of pre- versus post-treatment immune marker expression..

bCC-001			bCC-002			sCC-003		
Target name	Log2 fold change	-log10 pvalue	Target name	Log2 fold change	-log10 pvalue	Target name	Log2 fold change	-log10 pvalue
CD11c	2.310	1.125	VISTA	0.786	1.797	CD8	2.932	1.974
GITR	1.544	0.891	CD68	0.328	0.294	OX40L	1.870	1.833
OX40L	1.534	1.304	GITR	0.229	0.155	CD45	1.809	1.000
PD-1	1.484	1.257	CD45	0.008	0.161	CD4	1.806	0.955
GZMB	1.472	1.320	Beta-2-microglobulin	-0.044	0.086	GZMB	1.774	2.347
B7-H3	1.366	1.917	LAG3	-0.090	0.151	CD11c	1.667	0.865
LAG3	1.270	0.663	Fibronectin	-0.215	0.208	CD3	1.658	1.119
ARG1	1.214	0.501	CD8	-0.296	0.171	B7-H3	1.557	1.118
CD68	1.165	0.916	CD56	-0.337	0.523	CD56	1.317	0.735
CD4	0.952	0.636	STING	-0.340	0.288	ARG1	1.261	1.783
4-1BB	0.909	1.305	CD4	-0.367	0.140	CTLA4	1.211	1.092
CD8	0.907	0.433	PD-1	-0.414	0.209	HLA-DR	1.174	0.840
CD56	0.898	0.681	CD20	-0.416	0.306	SMA	1.147	0.837
Tim-3	0.898	0.913	PD-L1	-0.464	0.045	CD20	1.043	1.408
VISTA	0.844	0.604	OX40L	-0.521	0.747	Tim-3	1.011	0.758
Fibronectin	0.716	0.568	CD3	-0.528	0.271	VISTA	0.877	1.107
Beta-2-microglobulin	0.679	0.560	B7-H3	-0.654	0.752	CD68	0.815	0.954
PD-L1	0.654	0.570	GZMB	-0.700	0.505	Beta-2-microglobulin	0.784	1.137
STING	0.641	0.422	IDO1	-0.729	0.088	GITR	0.760	0.558
CD45	0.596	0.430	HLA-DR	-0.744	0.105	STING	0.407	0.537
HLA-DR	0.532	0.295	Tim-3	-0.825	1.923	Fibronectin	0.388	0.240
PanCk	0.514	0.625	PanCk	-0.898	0.524	4-1BB	0.268	0.991
CTLA4	0.466	0.091	ARG1	-1.022	1.320	PD-L1	0.221	0.010
SMA	0.405	0.798	Ki-67	-1.048	0.888	PD-1	0.098	0.307
Ki-67	0.118	0.170	SMA	-1.070	1.579	IDO1	0.061	0.156
CD20	0.077	0.292	4-1BB	-1.070	1.100	Ki-67	-0.083	0.168
CD3	0.048	0.178	CTLA4	-1.283	1.407	LAG3	-0.543	0.614
IDO1	-0.373	0.287	CD11c	-1.295	0.429	PanCk	-0.574	0.427

bCC-004			Mel-006			Mel-008		
Target name	Log2 fold change	-log10 pvalue	Target name	Log2 fold change	-log10 pvalue	Target name	Log2 fold change	-log10 pvalue
ARG1	2.161	1.633	Ki-67	1.141	3.250	PanCk	2.394	0.963
4-1BB	1.979	0.770	GZMB	0.057	0.151	Fibronectin	1.823	2.502
IDO1	1.850	0.638	CD45	0.004	0.079	B7-H3	1.365	3.920
CD11c	1.733	2.641	CD8	-0.030	0.039	Beta-2-microglobulin	1.026	4.192
PD-L1	1.678	1.502	VISTA	-0.128	0.313	CTLA4	0.873	0.919
Fibronectin	1.627	1.451	CD56	-0.182	0.421	CD4	0.862	1.503
CD56	1.513	1.186	CD3	-0.258	0.351	SMA	0.843	1.739
CTLA4	1.469	0.808	PD-1	-0.352	1.280	CD3	0.752	1.248
Beta-2-microglobulin	1.420	3.774	CD11c	-0.386	0.981	CD8	0.744	1.139
CD45	1.372	2.412	Beta-2-microglobulin	-0.432	2.552	CD45	0.572	1.006
GITR	1.354	0.673	STING	-0.443	1.259	HLA-DR	0.433	0.601
CD3	1.323	1.510	CD20	-0.471	1.546	CD20	0.091	0.179
HLA-DR	1.112	0.772	LAG3	-0.473	0.974	GITR	0.075	0.002
Tim-3	1.109	1.541	SMA	-0.475	1.029	PD-1	-0.033	0.087
OX40L	1.093	0.483	IDO1	-0.488	0.507	4-1BB	-0.133	0.326
CD4	1.015	1.406	GITR	-0.563	0.672	LAG3	-0.259	0.693
B7-H3	0.957	1.759	CD4	-0.564	1.007	STING	-0.273	0.585
CD20	0.781	0.526	Tim-3	-0.619	1.595	CD56	-0.362	1.087
VISTA	0.699	0.754	OX40L	-0.683	1.450	CD11c	-0.410	0.839
CD8	0.699	0.596	CTLA4	-0.684	0.062	OX40L	-0.560	1.555
Ki-67	0.682	0.985	PanCk	-0.695	0.817	ARG1	-0.601	1.817
LAG3	0.617	0.306	HLA-DR	-0.768	3.024	GZMB	-0.602	1.512
PD-1	0.570	0.943	PD-L1	-0.772	1.454	Tim-3	-0.610	1.533
CD68	0.522	0.743	ARG1	-1.096	2.402	CD68	-0.625	1.502
STING	0.496	1.143	B7-H3	-1.161	2.366	PD-L1	-0.751	1.558
PanCk	0.447	0.881	CD68	-1.295	2.389	VISTA	-1.018	2.634
SMA	0.212	0.284	4-1BB	-1.314	2.763	Ki-67	-2.125	6.431
GZMB	-1.294	0.870	Fibronectin	-2.191	2.676	IDO1	-2.904	2.855

Supplementary Tables

Supplementary Table 5. GeoMx analysis data of epithelial markers versus immune markers in post-treatment tissue.

bCC-001			bCC-002			sCC-003			bCC-004			Mel-006			Mel-008		
**Target name**	**Log2 fold change**	**-log10 pvalue**	**Target name**	**Log2 fold change**	**-log10 pvalue**	**Target name**	**Log2 fold change**	**-log10 pvalue**	**Target name**	**Log2 fold change**	**-log10 pvalue**	**Target name**	**Log2 fold change**	**-log10 pvalue**	**Target name**	**Log2 fold change**	**-log10 pvalue**
CD45	3.624	1.071	VISTA	3.604	2.029	CD3	2.096	1.092	CD45	2.365	1.734	CD3	3.679	2.190	GZMB	n/a	n/a
VISTA	2.788	1.388	CD45	2.748	1.912	CD8	1.823	1.104	CD3	1.887	1.183	CD45	3.664	4.049	PD-1	n/a	n/a
HLA-DR	2.738	0.969	HLA-DR	2.151	1.065	CD4	1.691	2.147	CD8	1.563	0.941	Ki-67	3.116	3.888	CD45	n/a	n/a
CD4	2.341	1.014	CD11c	1.930	1.051	Fibronectin	1.463	1.740	VISTA	1.292	0.629	IDO1	3.001	1.620	CD3	n/a	n/a
CD3	1.877	0.836	IDO1	1.862	0.844	CD45	1.450	1.223	IDO1	1.272	0.816	CD8	2.985	2.455	PD-L1	n/a	n/a
CD8	1.756	0.738	CD3	1.708	1.076	ARG1	1.388	0.900	HLA-DR	1.188	1.084	CD4	2.728	2.202	SMA	n/a	n/a
Tim-3	1.751	1.364	CD4	1.437	0.977	VISTA	1.264	1.150	CD4	1.166	1.212	VISTA	2.277	3.321	Ki-67	n/a	n/a
CD11c	1.655	1.020	CD68	1.279	1.031	HLA-DR	1.112	2.582	CD11c	0.977	0.835	HLA-DR	1.902	4.584	CD68	n/a	n/a
GITR	1.453	1.395	Beta-2-microglobulin	1.234	1.029	4-1BB	0.915	0.714	Fibronectin	0.790	0.685	CD11c	1.673	3.272	CD11c	n/a	n/a
CD68	1.136	1.069	ARG1	1.213	0.867	CTLA4	0.790	0.982	CD68	0.635	0.565	CD56	1.031	2.381	CD56	n/a	n/a
IDO1	1.026	0.667	4-1BB	1.177	1.163	STING	0.445	0.322	CTLA4	0.627	2.024	STING	0.795	1.882	PanCk	n/a	n/a
LAG3	0.850	0.610	CD20	1.117	0.414	IDO1	0.427	0.467	LAG3	0.513	0.238	CD68	0.534	0.661	HLA-DR	n/a	n/a
STING	0.770	0.578	STING	1.113	0.676	GITR	0.404	0.334	GITR	0.437	0.962	CD20	0.387	0.809	CD20	n/a	n/a
4-1BB	0.608	0.438	Tim-3	1.058	1.260	CD11c	0.363	0.229	PD-1	0.311	0.008	GZMB	0.384	1.071	Beta-2-microglobulin	n/a	n/a
Fibronectin	0.478	0.507	PD-L1	1.054	0.711	CD68	0.327	0.487	Beta-2-microglobulin	0.048	0.137	GITR	0.231	0.193	CTLA4	n/a	n/a
OX40L	0.429	0.373	SMA	1.009	0.835	CD20	0.221	0.103	PD-L1	-0.112	0.018	PD-1	0.186	0.741	CD8	n/a	n/a
SMA	0.348	0.226	GITR	0.998	0.964	GZMB	0.204	0.284	4-1BB	-0.165	0.170	SMA	-0.066	0.099	Fibronectin	n/a	n/a
PD-L1	0.342	0.200	CTLA4	0.983	0.899	SMA	0.139	0.189	ARG1	-0.269	0.225	Fibronectin	-0.160	0.134	CD4	n/a	n/a
ARG1	0.183	0.252	Fibronectin	0.959	1.060	CD56	0.111	0.253	OX40L	-0.357	0.265	LAG3	-0.170	0.211	LAG3	n/a	n/a
Beta-2-microglobulin	-0.182	0.014	CD8	0.903	0.385	Tim-3	-0.306	0.160	Tim-3	-0.395	0.228	OX40L	-0.402	0.711	4-1BB	n/a	n/a
B7-H3	-0.336	0.280	LAG3	0.615	0.252	PD-1	-0.310	0.024	CD20	-0.474	0.239	4-1BB	-0.443	0.434	B7-H3	n/a	n/a
PD-1	-0.519	0.013	PD-1	0.058	0.098	B7-H3	-0.457	0.441	SMA	-0.556	0.426	Tim-3	-0.460	0.661	Tim-3	n/a	n/a
CTLA4	-0.542	0.353	CD56	-0.028	0.082	PD-L1	-0.497	0.433	STING	-0.850	0.954	ARG1	-0.556	1.225	OX40L	n/a	n/a
CD20	-0.578	0.448	GZMB	-0.848	0.644	Beta-2-microglobulin	-0.648	0.720	GZMB	-1.795	1.810	CTLA4	-1.018	0.390	GITR	n/a	n/a
GZMB	-0.948	1.498	B7-H3	-0.924	1.051	LAG3	-0.799	0.636	Ki-67	-2.001	1.268	Beta-2-microglobulin	-1.066	0.962	STING	n/a	n/a
PanCk	-2.770	1.498	OX40L	-1.320	1.218	OX40L	-1.309	0.648	B7-H3	-2.389	1.937	PanCk	-1.204	0.704	IDO1	n/a	n/a
Ki-67	-4.441	2.965	Ki-67	-1.802	1.289	Ki-67	-2.429	2.239	CD56	-3.879	1.370	PD-L1	-1.573	1.540	ARG1	n/a	n/a
CD56	-4.789	1.923	PanCk	-2.665	1.631	PanCk	-5.054	2.229	PanCk	-4.788	1.530	B7-H3	-3.340	1.573	VISTA	n/a	n/a

Supplementary Figures

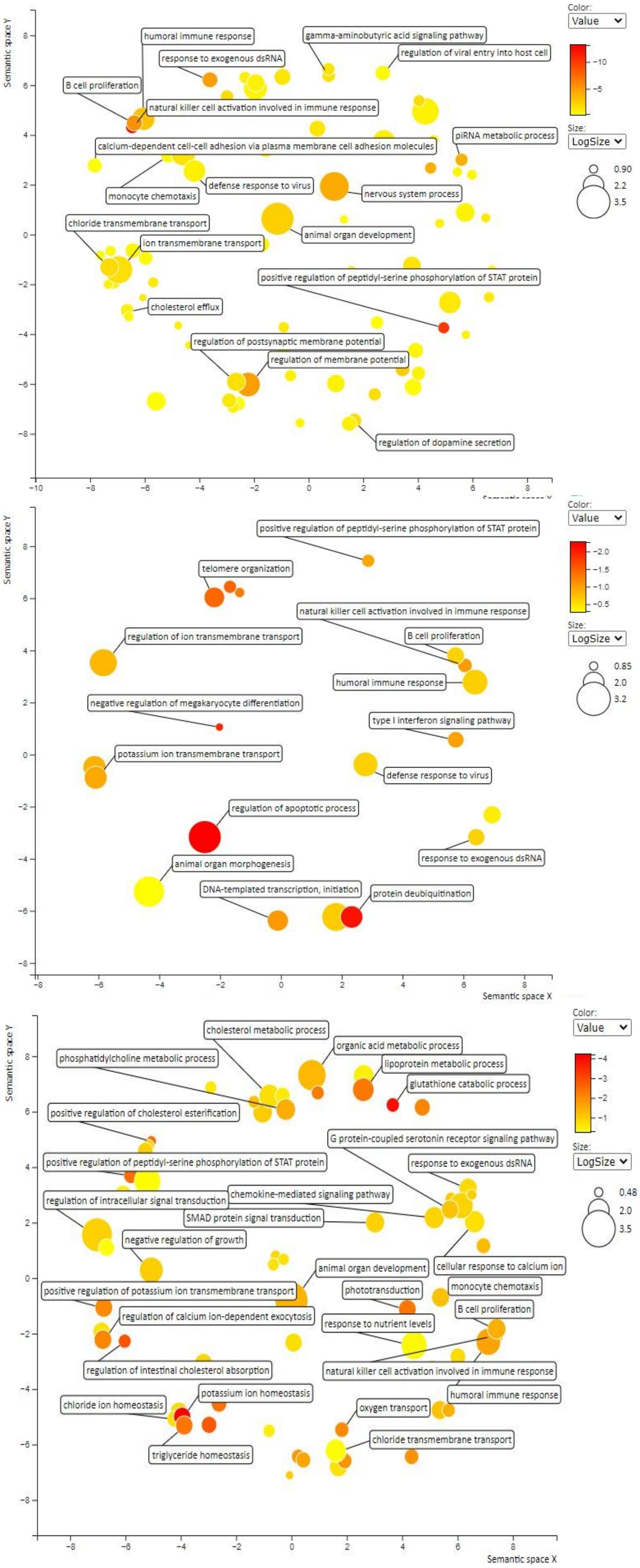

Supplementary Figure 1. Gene ontology (GO) plot representing RNA sequence analysis from patients 1, 2, and 3. The axes in the plot have no intrinsic meaning. Revigo uses Multidimensional Scaling (MDS) to reduce the dimensionality of a matrix of the GO terms pairwise semantic similarities. The resulting projection may be highly non linear. The guiding principle is that semantically similar GO terms should remain close together in the plot. Repeated runs of Revigo may yield different arrangements, but the term distances remain similar.

Supplementary Figures

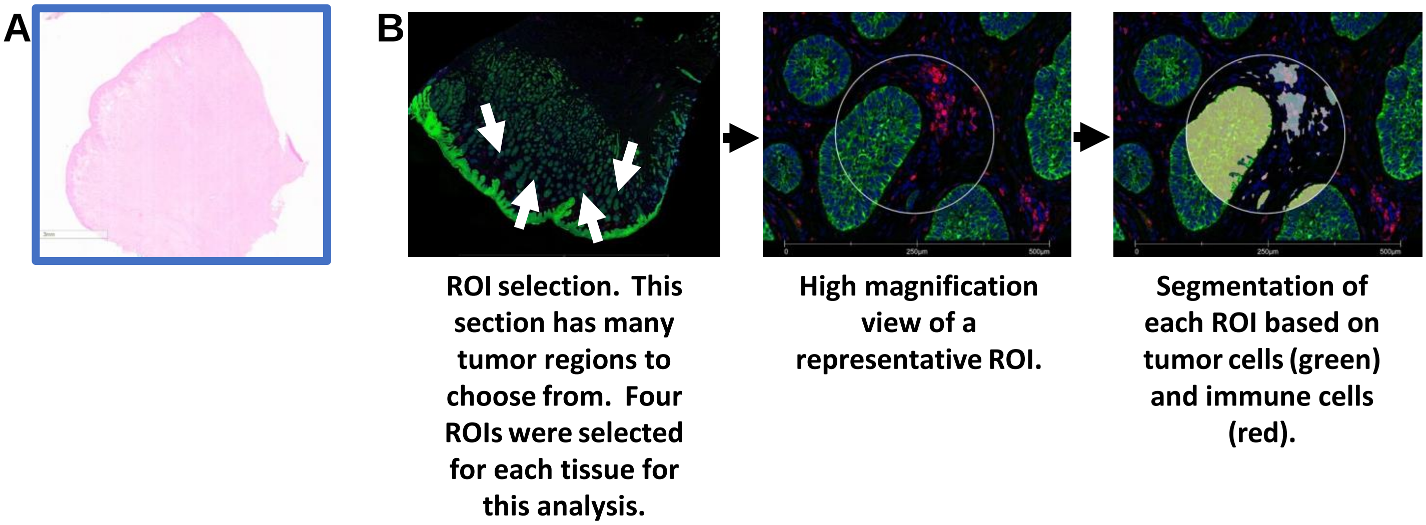

Supplementary Figure 2. Workflow used for digital spatial profiling of clinical samples. A) Pathologist review of an H&E-stained slide used to verify presence of tumor cells within biopsy. B) An unstained serial section was then labeled overnight with a cocktail of non-fluorescent antibodies (from the GeoMx Protein Core and IO panels) conjugated to the GeoMx DSP barcodes and fluorescent antibodies against panCK (basal or squamous cell carcinoma samples) or MART1 (melanoma samples) and CD45 to identify epithelial cells and immune cells, respectively; Syto 13 dye was used to label nuclei. Regions of interest (ROI) were selected using the morphology markers as guides. The ROIs were segmented into tumor cells and immune cells followed by independent collection of their corresponding DSP barcodes by the GeoMx instrument. The DSP barcodes were processed using NanoString's nCounter protocol to yield expression data for 28 study proteins, 3 positive controls, and 3 negative controls covering the two panels).

Supplementary Figures

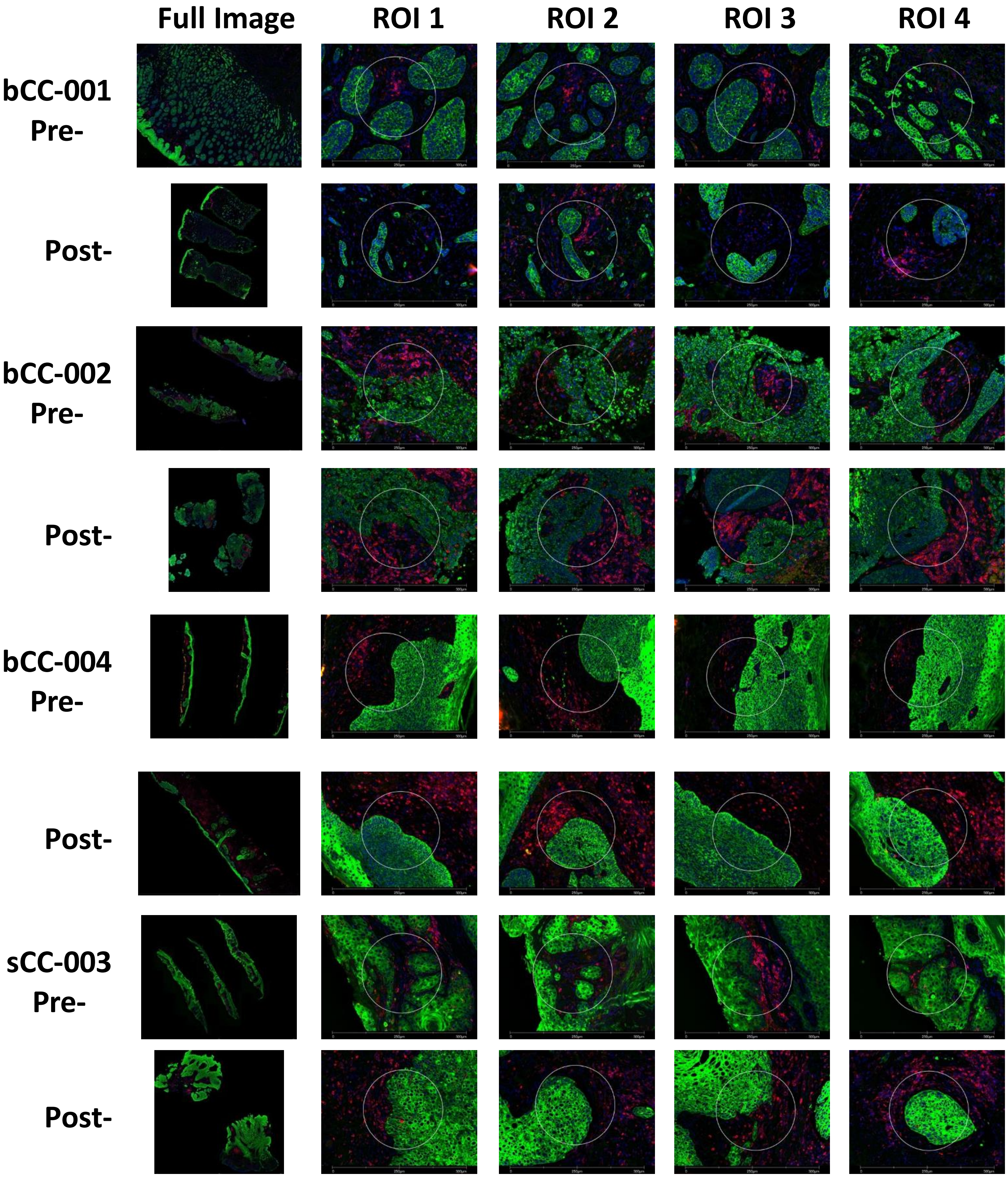

Supplementary Figure 3A. Workflow used for digital spatial profiling of clinical samples. A) Pathologist review of an H&E-stained slide used to verify presence of tumor cells within biopsy. B) An unstained serial section was then labeled overnight with a cocktail of non-fluorescent antibodies (from the GeoMx Protein Core and IO panels) conjugated to the GeoMx DSP barcodes and fluorescent antibodies against panCK (basal or squamous cell carcinoma samples) or MART1 (melanoma samples) and CD45 to identify epithelial cells and immune cells, respectively; Syto 13 dye was used to label nuclei. Regions of interest (ROI) were selected using the morphology markers as guides. The ROIs were segmented into tumor cells and immune cells followed by independent collection of their corresponding

Supplementary Figures

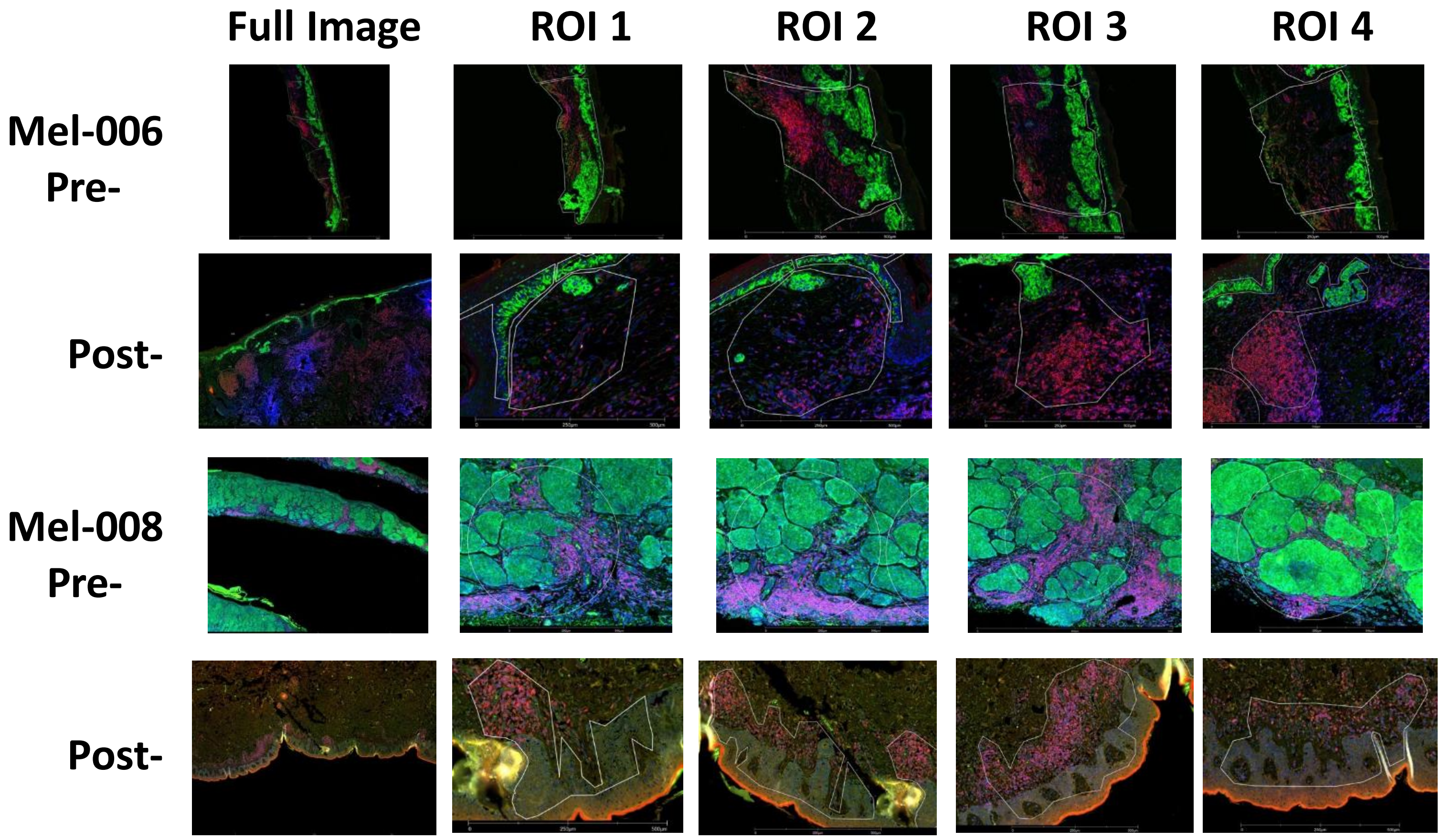

Supplementary Figure 3B. Representative GeoMx images of pre- and posttreatment study samples. Full image - low magnification of available tissue biopsies (first column). Four regions of interest were selected for each sample for analysis and from where DNA barcodes were cleaved and collected are shown by a circle in each image (the last 4 columns). Images of A) basal and squamous cell carcinoma tissue samples; and B) melanoma tissue samples. Pan-cytokeratin (green, epithelium, panel A); MART1 (green, epithelium, panel B), CD45 (red, immune cells, both panels A & B); Syto13 (blue, DNA, both panels A & B).

Supplementary Figures

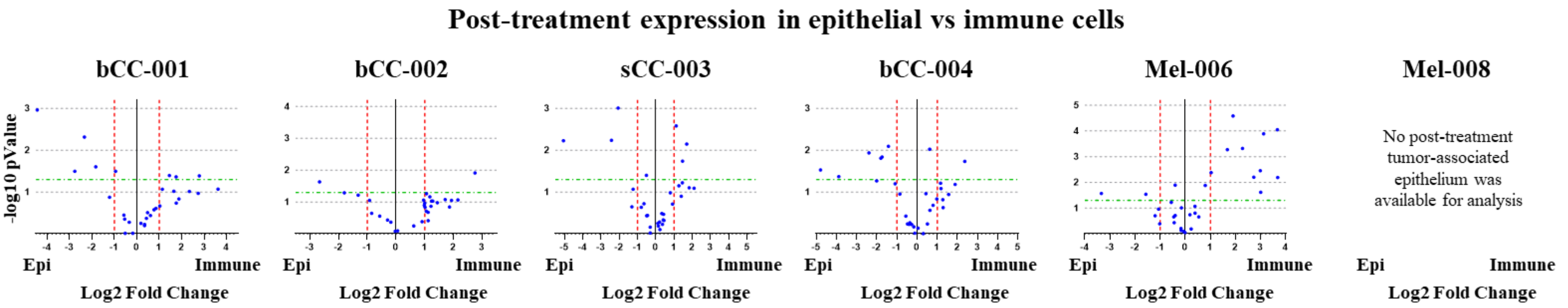

Supplementary Figure 4. Volcano plots showing differential protein expression. Expression was considered statistically significant if changes were ≥ ± 2-fold (≥ ± 1-fold in Log2 scale, red dashed vertical lines) with a p value ≤ 0.05 (≥ 1.3 in -Log10 scale, green dashed horizonal line). A paired t-test was used for these analyses. Comparison of post-treatment expression between epithelial and immune cells. An Excel file is provided for all volcano plots in Supplementary Table 5.
